# Governing AI in Mental Health: 50-State Legislative Review

**DOI:** 10.2196/80739

**Published:** 2025-10-31

**Authors:** J Nicholas Shumate, Eden Rozenblit, Matthew Flathers, Carlos A Larrauri, Christine Hau, Winna Xia, E Nicholas Torous, John Torous

**Affiliations:** 1 Division of Digital Psychiatry, Department of Psychiatry, Beth Israel Deaconess Medical Center, 330 Brookline Avenue, Rabb-2, Boston, 02215, United States, 1 617-667-6700 ext 11067; 2 Harvard T.H. Chan School of Public Health, Boston, MA, United States

**Keywords:** artificial intelligence, MH-AI, large language model, chatbot, LLM, machine learning, malpractice, liability, law, legislation, AI regulation, digital therapy, clinical oversight, generative AI, healthcare policy, technology ethics, digital health, legislative trends, policy analysis, regulatory gaps, health law, algorithmic bias, AI safety, AI risk, psychiatric tools, digital treatments, therapy bots, digital companions, medical regulation, AI oversight, mental health–related artificial intelligence

## Abstract

**Background:**

Mental health–related artificial intelligence (MH-AI) systems are proliferating across consumer and clinical contexts, outpacing regulatory frameworks and raising urgent questions about safety, accountability, and clinical integration. Reports of adverse events, including instances of self-harm and harmful clinical advice, highlight the risks of deploying such tools without clear standards and oversight. Federal authority over MH-AI is fragmented, leaving state legislatures to serve as de facto laboratories for MH-AI policy. Some states have been highly active in this area during recent legislative sessions. Yet, clinicians and professional organizations have mainly remained absent or sidelined from public commentary and policymaking bodies, raising concerns that new laws may diverge from the realities of mental health care.

**Objective:**

To systematically analyze recent state-level legislation relevant to MH-AI, categorize bills by relevance to mental health, identify major regulatory themes and gaps, and evaluate implications for clinicians and patients.

**Methods:**

We conducted a systematic analysis of bills introduced in all 50 US states between January 1, 2022, and May 19, 2025, using standardized searches on the legislative research website (LegiScan). Bills were screened and categorized using a custom 4-tier taxonomy based on their applicability to MH-AI. Bills passing threshold review were coded by topic using a 25-tag system developed through iterative consensus. Legally trained reviewers adjudicated final classifications to ensure consistency and rigor.

**Results:**

Among 793 state bills reviewed, 143 were identified as potentially impactful to MH-AI: 28 explicitly referenced mental health uses, while 115 had substantial or indirect implications. Of these 143 bills, 20 were enacted across 11 states. Legislative efforts varied widely, but 4 thematic domains consistently emerged: (1) professional oversight, including deployer liability and licensure obligations; (2) harm prevention, encompassing safety protocols, malpractice exposure, and risk stratification frameworks; (3) patient autonomy, particularly in areas of disclosure, consent, and transparency; and (4) data governance, with notable gaps in privacy protections for sensitive mental health data.

**Conclusions:**

State legislatures are rapidly shaping the regulatory landscape for MH-AI, but most laws treat mental health as incidental to broader artificial intelligence or health care regulation. Explicit mental health provisions remain rare, and clinician and patient perspectives are seldom incorporated into policymaking. The result is a fragmented and uneven environment that risks leaving patients unprotected and clinicians overburdened. Mental health professionals must proactively engage with legislators, professional organizations, and patient advocates to ensure that emerging frameworks address oversight, harm, autonomy, and privacy in ways that are clinically realistic, ethically sound, and supportive of flexible—but responsible—innovation.

## Introduction

The use of mental health–related artificial intelligence (MH-AI) is rapidly expanding, both in consumer and clinical domains. In this paper, MH-AI is broadly defined as any artificial intelligence (AI) system used in the delivery, facilitation, or simulation of mental health services. In April 2025, the Harvard Business Review reported that therapy and companionship had become the most frequently cited use cases for generative AI systems mentioned online [1]. Tens of millions of individuals are already engaging with AI systems as human-like companions, so-called “emotional support tools,” to ask mental health-related questions, or even as stand-in therapists [2-4]. On the clinical side, in one study, 33% (45/138) of psychiatrists recently reported using OpenAI’s ChatGPT to assist with clinical care, and—in the same study—75% (104/138) believed that patients are likely to consult generative AI before seeking a medical provider [5]. Practitioners, health systems, researchers, and other stakeholders are increasingly exploring the use of AI in various applications, including administering mood scales, diagnosis and treatment, risk stratification, administrative support, drug design, and the detection and monitoring of severe mental illnesses, with varying degrees of success [4,6-8].

These advances, however, are not without risk. While the authors identified no systematic review of harms related to MH-AI, numerous high-profile reports in the media of troubling adverse events—including alleged suicidal [9,10] and homicidal [11] acts—have been documented, contributing to the development of a dedicated “AI Incident Database” aimed at raising public awareness of the potential dangers of AI [12]. Further illustrating the risks, the Center for Countering Digital Hate recently found that generative AI tools produced harmful eating disorder information in 41% (74/180) of reviewed samples, such as recommending hiding uneaten food from parents and, with even the most restrictive AI tested, offering the advice of swallowing a tapeworm egg to lose weight under certain easily achieved conditions [13]. Such examples highlight how easily MH-AI tools can shift from supportive to harmful, particularly when deployed without clear standards, safeguards, or human oversight.

Multiple federal agencies and laws, including the Food and Drug Administration, Federal Trade Commission, Department of Health and Human Services, and the Health Insurance Portability and Accountability Act (HIPAA), exercise limited oversight over MH-AI technologies. However, federal authority in this space is constrained by the specific mandates and domains of each agency or statute, a fuller discussion of which can be found in studies by Kahane et al [14,15]. As a result, many MH-AI tools fall into regulatory gray zones or remain exempt from rules governing other medical technologies, particularly so-called “wellness” products and other technologies not specifically marketed as medical or therapy products [7,14-17]. In the absence of a comprehensive federal framework, there is little unified guidance for states or stakeholders. Consequently, states have become de facto policy laboratories, with state legislatures proposing hundreds of AI-related bills since 2022.

Despite the growing legislative interest in AI, public commentary from clinicians on specific proposed state MH-AI laws remains muted. Even major professional organizations, such as the American Psychiatric Association and the American Psychological Association, have issued mainly limited position statements, stopping short of offering detailed practice guidelines or public policy recommendations [16,17]. This absence of public clinical input is troubling given the complexity of regulating mental health care, where risks to patient safety, autonomy, professional ethics, and standards of care are profound. As explored throughout this paper, most proposed and enacted state laws lack meaningful integration of clinical insight and fail to address mental health use cases, thereby raising the risk that future norms (both regulatory and clinical) will be misaligned with the realities of patient care. In response, this paper synthesizes findings from a comprehensive review of state-level MH-AI legislation to identify emerging legislative trends and crucial gaps, as well as highlights the urgent need for clinician and patient engagement in shaping the future of MH-AI policy.

## Methods

The authors conducted a systematic analysis of state legislative activity across all 50 US states between January 1, 2022, and May 19, 2025, to identify bills relevant to the intersection of AI and mental health. The year 2022 was chosen as the start of the range due to both the lack of significant bills related to MH-AI before this year on initial review and the first public release of OpenAI’s ChatGPT in November 2022, which marked a turning point in legislative activity on this front. The search was conducted on May 19, 2025. The search was limited to this date to allow the reviewers to conduct a cross-sectional analysis of bills without needing to account for changing bill status. This search was conducted on the LegiScan website to ensure that querying methods were consistent across states, regardless of variations in individual legislative website design and search functionality [18]. Boolean search queries that paired AI-related terms with mental health-related terms were used in order to narrow results to bills plausibly implicating MH-AI, while excluding bills referencing AI or mental health in isolation and unrelated contexts. The following process was used: (1) navigate to LegiScan, (2) set sessions to “all,” and (3) for each state, the following query was used: intro:20220101..20250519 AND (“artificial intelligence” OR “predictive model” OR chatbot OR LLM OR “language model” OR “machine learning” OR “deep learning”) AND (mental OR behavioral OR psych OR healthcare OR “health care”).

Each retrieved bill was screened for initial relevance by 1 of 4 authors, each responsible for approximately a quarter of the bills. Furthermore, 2 authors were legally trained (JNS and CAL), and 2 had undergone multiple rounds of training to achieve rating reliability (ER and MF). All bills were then reviewed a second time by the first author (JNS), also a legally trained individual, to make a final adjudication. Bills determined to have no plausible bearing on MH-AI were coded as Not Relevant (NR) and excluded from further analysis. Companion bills with versions in both state legislative chambers and bills continued from a previous legislative session were excluded if a more recent version was available.

Bills passing the threshold screen were then independently reviewed by 2 research assistants (ER and MF) using a 4-tiered coding taxonomy developed by the study team (Table 1). This taxonomy distinguished bills based on the specificity and relevance of their relationship to MH-AI systems and clinical practice. A legally trained researcher (CAL) reviewed these codings and provided an opinion on any that lacked complete consensus. Any bills that received consensus from the majority of reviewers were labeled accordingly. The first author (JNS) reviewed all bills without a majority consensus and made a final determination for taxonomy coding purposes.

Following taxonomy coding, each of the remaining bills was independently reviewed by 2 research assistants (CH and WX) using a set of 25 predefined topic tags, as described in Table 2. These tags were developed through an iterative consensus process based on terms and provisions found in the collected bills during the initial relevance threshold review. Tags were selected for their topical significance to the intersection of AI and mental health. Tag assignment was descriptive rather than qualitative, signaling only that the tag’s specific topic was addressed in the bill in some form. A second pair of research assistants (ER and MF) subsequently reviewed and reconciled any discrepancies in tagging. Final review and quality control were conducted by a legally trained member of the research team to ensure consistency and interpretive rigor.

**Table 1.** Four-tier coding taxonomy for classifying state artificial intelligence bills by relevance to mental health applications, with code labels, definitions, and inclusion criteria.

Code	Category	Definition	Inclusion criteria
E^a^	Explicit	Bills that explicitly reference mental health, behavioral health, psychotherapy, or related services in the context of AI^b^ development, regulation, or application.	The bill directly names mental health uses of AI, specific clinical applications, or mental health contexts as targets of regulation, policy, or oversight.
SR^c^	Substantively relevant	Bills that govern MH-AI^d^ in ways that have direct, foreseeable implications for mental health services or stakeholders, even if mental health is not explicitly or substantively discussed in the bill’s text.	The bill regulates MH-AI in a way that predictably impacts mental health uses, delivery, or providers, regardless of whether mental health is explicitly mentioned.
II^e^	Incidentally implicative	Bills that are broadly written and might include MH-AI, but only in a general or indirect way. Clinical impact is uncertain or minimal.	MH-AI falls or could fall under the bill’s scope, but direct mechanisms or practical effects at the clinical level are not apparent or minimal.
NR^f^	Not relevant	Bills with no meaningful relationship to MH-AI services, even under expansive interpretations.	The bill does not touch on mental health services.

a E: explicit.

b AI: artificial intelligence.

c SR: substantively relevant.

d MH-AI: mental health–related artificial intelligence.

e II: incidentally implicative.

f NR: not relevant.

**Table 2.** Definitions of the 25 topic tags and their frequency among included mental health–related artificial intelligence bills (n=143); counts indicate presence of a topic, not strength or quality.

Tag	Number of bills	Definition
Civil penalties	100	Applies any kind of civil penalty to violators (eg, noncriminal penalties such as system suspension, civil fines, creation of private right of action to sue, profit disgorgement, suspension of noncompliant systems, punitive monitoring, or application of a separate civil enforcement statute).
Disclosure or consent	96	Implements any requirement to disclose use or features of the AI^a^ system (including disclaimers), consent to the use of such systems or features, or the ability to revoke consent.
Transparency	90	Implements requirements involving public or patient rights to access AI system data (eg, requests to obtain data, public inventories of AI systems, publication, or transparency requirements).
Consumer protection	84	Provisions concerning fraudulent, manipulative, or deceptive use of MH-AI^b^ systems, including in advertising.
Monitoring	80	Applies any kind of monitoring requirements for MH-AI (eg, live supervision, submission to audits or documentation processes, generation of reports, or postmarket surveillance).
Data protection	77	Implements any requirements for data privacy, data security, or data retention or deletion (eg, encryption requirements, secure storage, or data purging policies).
Discrimination or bias	68	Implements any requirement regarding discrimination, bias, or fairness.
Postmarket review	66	Implements requirements for any level of scheduled or routine review after the AI product has been marketed or implemented (eg, postmarket surveillance, auditing, risk assessments, and efficacy reviews), subject to regulatory oversight.
Safety standards	50	Pertains to safety standards for MH-AI (eg, human overrides, emergency protocols, or prohibitions on high-risk uses) or safety-based exceptions to other requirements (eg, bypassing procedures when delays risk harm and allowing immediate protective actions).
Vulnerable populations	50	Creates any responsibilities related to vulnerable populations (eg, older adults, children, disabled, and foreign-language speakers), such as mandated reporting requirements, accessibility requirements, or parental controls.
Human-in-the-loop	49	Explicitly requires a human to monitor, approve, or participate in an essential part of the provision of the MH-AI service.
Meta or biometric data	46	Regulates biometric data, behavioral data, or metadata used by MH-AI systems.
Research	45	Pertains to or would affect mental health research (eg, data collection, consent requirements, ethical guidelines, and exemptions for legitimate research use).
Practitioner responsibilities	37	Applies any kind of requirements on practitioners—or waivers or exemptions—related to their use of AI systems.
Risk classification	34	Implements or defines the scope of the law by a risk classification system (eg, “high risk” AI system, consequential decisions, and similar frameworks).
Premarket review	34	Implements requirements for any level of regulatory review before the AI product being offered or implemented (eg, state commission approval, FDA^c^ approval, and submission of risk assessments).
Event reporting	29	Creates a system for reporting adverse events, near misses, or other safety events involving MH-AI.
Special purpose entities	22	Creates or assigns committees, subcommittees, task forces, or similar special-purpose bodies pertaining to MH-AI.
Opt out	18	Provides for the ability to opt out of AI services in favor of receiving equivalent human-delivered health services.
Licensing board oversight	17	Applies any kind of oversight by state professional licensing boards (eg, requiring board approval of MH-AI systems used in diagnosis and treatment or allowing boards to discipline individuals or systems).
Malpractice or liability	16	Pertains to medical malpractice involving AI, including liability allocation for AI-related harm (eg, standards of evidence, assigning responsibility to deployers, developers, practitioners, or manufacturers, liability shields or limitations, and use of MH-AI records and data in litigation).
Criminal penalties	11	Applies any kind of criminal penalty to violators (eg, criminal fines, incarceration, and misdemeanor or felony designations).
Pilot or sandbox	11	Provides for regulatory pilot programs or sandbox systems, allowing AI products to be tested and receive feedback from regulators prior to full marketing.
Payments or insurance	10	Regulations on insurance coverage, reimbursement, and payment models.
Prescribing	2	Applies any requirements or waivers regarding prescriptions.

a AI: artificial intelligence.

b MH-AI: mental health–related artificial intelligence.

c FDA: Food and Drug Administration.

## Results

The search yielded 793 total bills, of which 523 were excluded after initial threshold review as NR, and 124 were excluded as companion bills or superseded versions. The 146 bills remaining underwent complete taxonomy coding. Following consensus determination and final review, 3 additional bills were recoded as NR, leaving 143 bills in the final analytic sample. Among these, 28 were classified as Explicit, 79 as Substantively Relevant (SR), and 36 as Incidentally Implicative (II). Notably, of these bills, 20 had been enacted into law at the time of this analysis.

**Figure 1.**
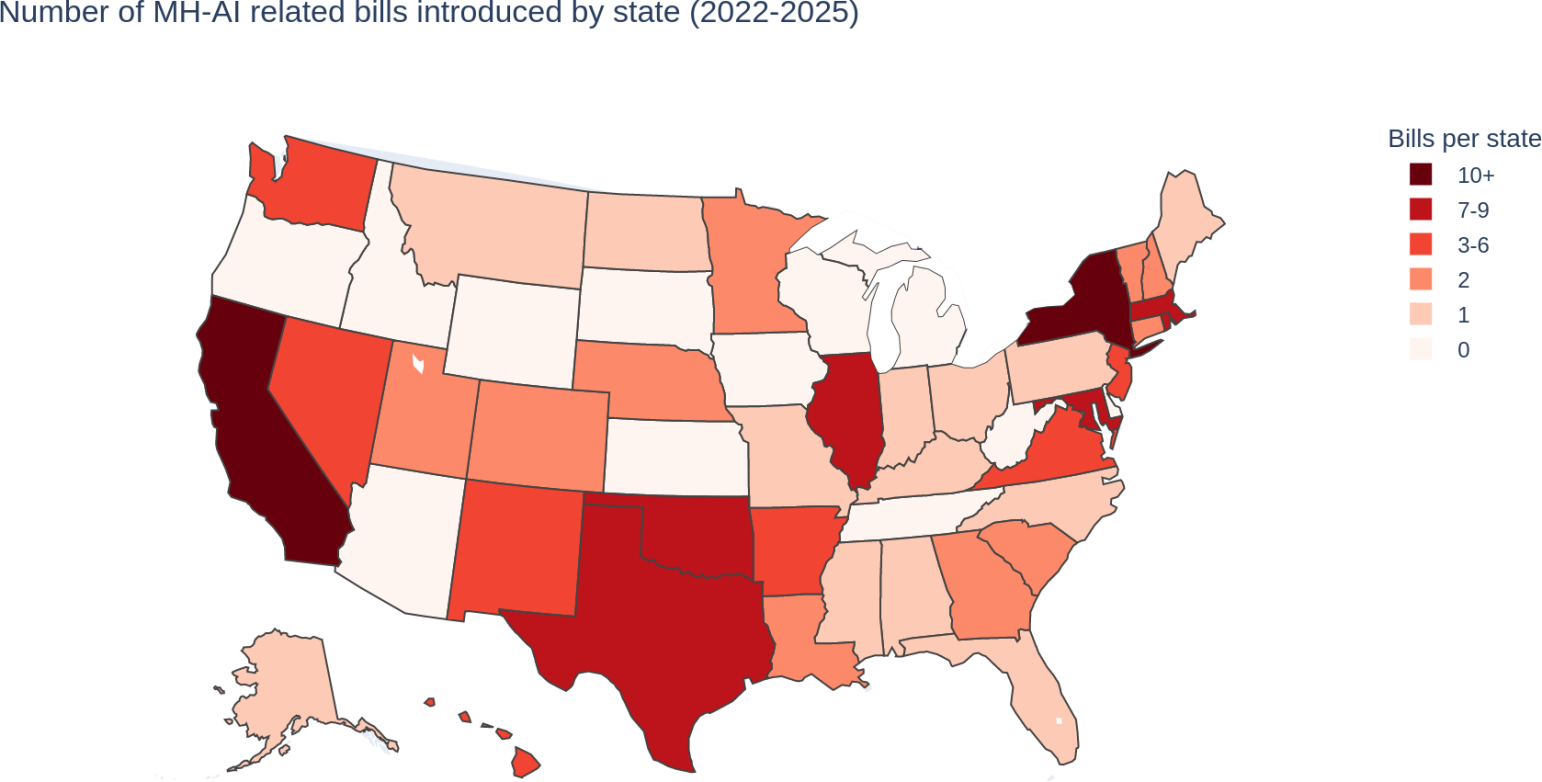
State counts of mental health–related artificial intelligence–relevant bills introduced January 1, 2022-May 19, 2025, displayed as a choropleth (bins: 0, 1, 2, 3‐6, 7‐9, 10+). Counts exclude not relevant bills and deduplicate companion or superseded versions. MH-AI: mental health–related artificial intelligence.

A state-by-state summary of the distribution of these codes has been illustrated in Figure 1 and Appendix S1 in Multimedia Appendix 1. Clear outliers include California with 19 bills meeting inclusion criteria and the 12 states with no bills meeting the inclusion criteria: Oregon, Michigan, Kansas, Tennessee, Idaho, Iowa, Delaware, Arizona, Wisconsin, West Virginia, Wyoming, and South Dakota.

**Figure 2.**
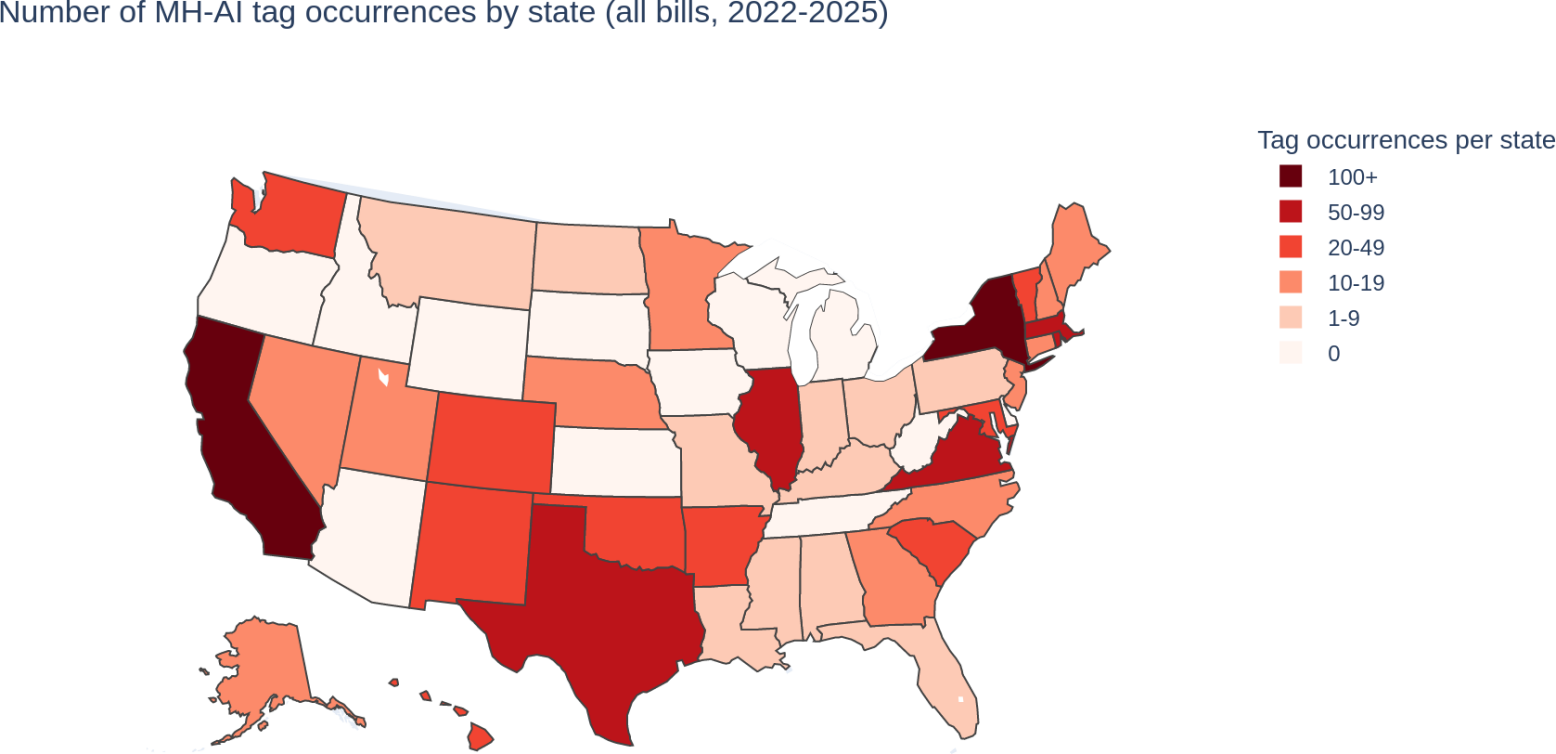
Total tag incidence by state. The sum of all tag occurrences across included bills (tags may appear in multiple bills; categories are nonmutually exclusive). Differences between tag density here and bill density in Figure 1 likely reflect individual bills that carry many tags, fragmented drafting that spreads topics across multiple narrower bills, and presence-based coding in which a single bill can receive multiple tags. MH-AI: mental health–related artificial intelligence.

**Figure 3.**
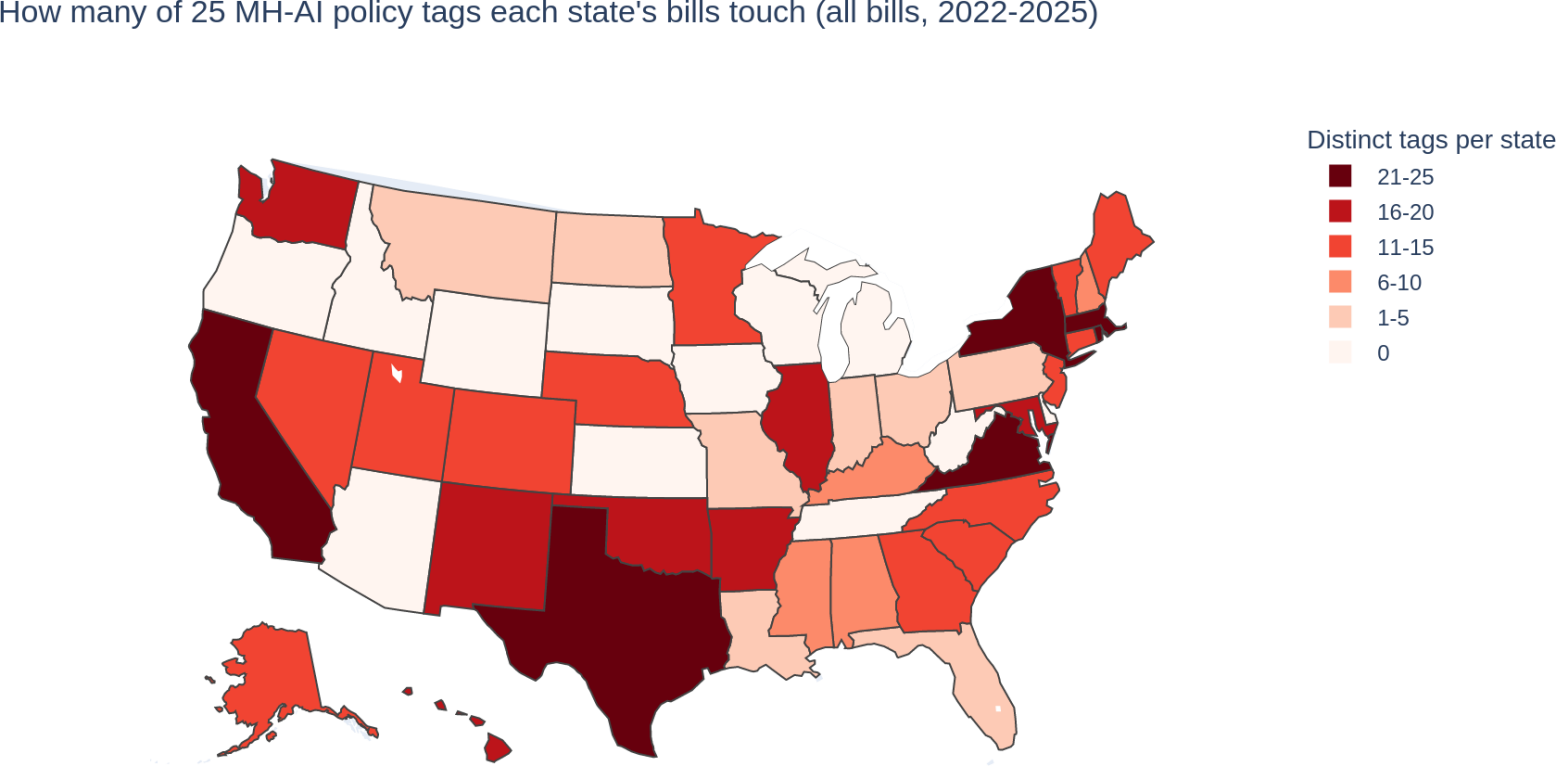
Tag breadth by state: the number of unique topic tags (out of 25) represented in included bills introduced January 1, 2022-May 19, 2025. Each tag is counted once per state regardless of how many bills contain it; not relevant and companion or superseded bills are excluded. Higher values indicate wider topical coverage, not the strength or effectiveness of a state’s mental health–related artificial intelligence framework (contrast with Figure 2’s tag incidence). MH-AI: mental health–related artificial intelligence.

Tagging across these 143 bills revealed instances of all 25 tags with variable incidence (Table 2 and Figures 2-3).

Individual analysis of taxonomy-coded bills and their tags revealed hundreds of variations of provisions potentially or explicitly impacting MH-AI. California, Texas, New York, and Virginia were notable outliers in the breadth of distinct tags addressed by their proposed laws. Several notable policy directions and gaps also appeared, as described in Table 3 (sorted into significant areas of legal and clinical relevance).

**Table 3.** Notable policy domains in mental health–related artificial intelligence state legislation: number of bills touching each domain (nonmutually exclusive; n=143).

Policy domain	Number of bills, n (%)
Professional oversight and responsibilities	
Explicit oversight by licensed mental health professionals required	6 (4.2)
Authorization for oversight by professional licensing boards	17 (11.9)
Governance and ethical standards	
Formation of special task forces, committees, or relevant entities with jurisdiction overlapping with MH-AI^a^	10 (7)
Ethical frameworks referenced to guide AI^b^ use	12 (8.39)
Alignment with National Institute of Standards and Technology (NIST) or other national best-practice standards	14 (9.79)
Consumer protection and enforcement	
Violations explicitly tied to existing state consumer protection laws	39 (27.27)
Explicit creation of private right of action for AI-induced harms	30 (20.98)
Assignment of punitive or super-compensatory damages or penalties	11 (7.69)
Violations tied directly to professional disciplinary actions	12 (8.39)
Allocation of malpractice liability or responsibility for MH-AI harms, including strict liability, immunities, and affirmative defenses	11 (7.69)
User protections and disclosures	
Explicit informed consent requirements for MH-AI use	4 (2.8)
Mandated disclosure of AI interactions, including continuous or repeated disclosures	33 (23.08)
Restrictions on AI systems simulating professional licensure or impersonating clinicians	6 (4.2)
Restrictions on advertising	14 (9.79)
Right to human review of AI-decision appeals	17 (11.89)
Allows for users to opt out of AI services in favor of human-provided services	9 (6.29)
Crisis response and safety	
Mandated crisis response planning for suicidal ideation or threats	8 (5.59)
Exemptions from certain requirements (eg, data privacy) when compliance could result in harm	15 (10.49)
Special populations and agency-specific provisions	
Specific protections designated for children	39 (27.27)
Application exclusively to state agencies and organizations	9 (6.29)
Transparency and accountability	
Public registry or inventory of AI systems mandated	19 (13.29)
Exemptions for entities or data covered under HIPAA^c^	22 (15.38)

a MH-AI: mental health–related artificial intelligence.

b AI: artificial intelligence.

c HIPAA: Health Insurance Portability and Accountability Act.

No attempt was made to analyze the variance between enacted bills and unenacted bills, as such analysis would likely be arbitrary and misleading when the data were collected midlegislative session. However, an additional table and figures compiling tags associated only with enacted bills are provided in Appendix S2 in Multimedia Appendix 1**,** offering an interesting point of comparison. In keeping with the most rigorous legal data transparency methods described by Pepin et al [19], all legal mapping methods are described herein, and all results were integrated into a single publicly available database discussed below.

## Discussion

### Principal Findings

Despite growing interest in the use of AI in health care, few state laws explicitly address its application in mental health. This analysis reveals a fragmented policy environment in which mental health–specific considerations are often overlooked or subsumed under broader AI or health care regulations. In the sections that follow, the authors examine trends in legislative activity and identify 4 key policy domains—oversight, harm, autonomy, and privacy—that warrant closer attention. These domains are likely to shape how enacted legislation is interpreted, implemented, and enforced. Illustrative examples of bill provisions are presented alongside a discussion of critical regulatory gaps. Together, these findings suggest the need for more targeted policymaking to ensure that MH-AI technologies are developed and deployed in a manner that protects patients and supports mental health professionals in delivering effective and innovative clinical care.

### Taxonomy Analysis

Of the 143 bills identified, only 28 explicitly address MH-AI, while 115 regulate MH-AI either substantially or incidentally. State-level patterns reveal an even sharper contrast. Only 13 states had proposed bills specifically mentioning mental health (or related terms), and, of these, only 3 had been enacted by the close of the study window. By comparison, 34 states had proposed laws that substantially or incidentally affected MH-AI regulation through provisions aimed at adjacent areas such as data privacy, general health care, or other regulatory domains. Of those, 17 were enacted. In total, only 2 states had enacted any explicit-coded laws from the sample (Utah [20] and New York [21]), 7 states enacted an SR-coded law, and 11 states enacted an II-coded law (Multimedia Appendix 1) at the time of data collection.

An explicit-coded bill example would include New Jersey’s Senate Bill (SB) 4463, which explicitly prohibits AI systems from being advertised as licensed mental health professionals [22]. An example of an SR-coded bill is Rhode Island’s SB 627, which aims to regulate “high-risk” AI systems making “consequential decisions,” including those related to health care, and likely encompasses MH-AI systems [23]. However, the bill does not explicitly mention mental health or tailor its scope for mental health–related systems or services. It is possible that Rhode Island case law or agency guidance already interprets “health care” broadly enough to include mental health; however, such a consideration falls outside the scope of this study. In contrast, Missouri’s House Bill (HB) 1462, an II-coded bill, establishes general legal definitions and liability rules for AI systems without addressing health care [24]. Although not designed with MH-AI in mind, its provisions could still influence the regulatory context in which these technologies operate.

This jumbled distribution of MH-AI legislation underscores significant disparities in regulatory attention and preparedness. States with little or no relevant legislation may be relying on outdated or overly broad frameworks ill-suited to address the unique risks of MH-AI tools [25,26]. Conversely, states enacting broad AI or health care laws without mental health–specific provisions may inadvertently leave gaps in oversight, particularly for technologies operating at the margins of existing legal and clinical frameworks. In either case, there is a strong argument that mental health practitioners should take an active role in shaping these rapidly evolving laws.

### Trends and Themes

A closer analysis of the sampled bills reveals substantial variation in how states regulate MH-AI, with most efforts reflecting fragmented provisions rather than cohesive regulatory frameworks. Despite this heterogeneity, at least 4 prominent thematic domains emerge that require clinicians’ attention. The following section organizes these themes and highlights illustrative examples that reveal consequential divergences in scope, emphasis, and implementation strategies relevant to MH-AI. Equally important, this paper also discusses some of the ways in which state legislation has failed to engage with MH-AI, leaving prominent gaps that carry implications for both practice and oversight.

### Oversight

This section examines how a subset of state legislation addresses clinician oversight in MH-AI, focusing on 3 key areas: requirements for professional supervision, the integration of AI use into licensure and disciplinary frameworks, and the role of licensing boards in regulating state MH-AI.

A notable subset of legislation directly ties the use of AI to clinician responsibilities and professional licensure. For example, bills in Oklahoma, Texas, Rhode Island, and Massachusetts propose real-time professional supervision or continuous monitoring of MH-AI services [27-31]. Similarly, bills in Illinois, Louisiana, and Nevada would require clinicians to verify AI-generated outputs used in care delivery or communications (eg, Illinois HB 5649, Illinois HB 1806, Louisiana HB 114, Louisiana HB 916, Nevada Assembly Bill [AB] 406) [32-36]. Some states propose less stringent human oversight, mandating availability on request rather than continuous intervention (eg, Texas HB 4455) [37].

Several bills explicitly link compliance failures to professional disciplinary actions, effectively integrating AI use into the existing licensure framework (eg, Rhode Island HB 6285, Illinois HB 5649) [30,32,33,36,38-47]. Meanwhile, states such as Louisiana (HB 916), Georgia (HB 887), and Illinois (SB 2259) involve professional licensing boards directly, either to promulgate MH-AI rules or to explicitly approve clinical use (Massachusetts HB 1974, Rhode Island HB 6285) [30,31,35,38,47]. California’s SB 813 takes a distinctive approach by proposing voluntary certification by multidisciplinary stakeholder groups, which may potentially include clinicians [48]. Such board-based oversight offers potential advantages, including clinically informed standards, regulatory flexibility, and alignment with existing safety and ethical frameworks.

However, shifting oversight to licensing boards or individual licensed professionals also raises important concerns. Licensed clinical professionals may lack the expertise and resources to validate algorithmic reliability or effectively detect bias, potentially exposing them to disproportionate liability (as exemplified by North Carolina HB 934, discussed below) [49]. In addition, laws targeting only licensed clinicians (whether directly or through their licensing bodies) risk creating regulatory gaps by leaving MH-AI use among unlicensed actors—such as life coaches, wellness influencers, peer support groups, and AI-driven self-help platforms—as well as AI system developers and deployers who fall outside traditional professional licensing requirements, underregulated. This could paradoxically compromise public safety while imposing undue and anticompetitive burdens on licensed practitioners (eg, Illinois SB 2259, Georgia HB 887, California SB 503, Arkansas HB 1816, Virginia HB 916; Texas SB 1188, Rhode Island HB 6285, Oklahoma HB 1915) [28,29,32,33,37,38,44,46,47,50-53]. If a developer or deployer can bypass meaningful oversight and safety regimes by marketing their product as a wellness tool or companion chatbot and avoid the labels used by a state’s laws, then the laws may both be ineffective and stifling of innovation in the professions most likely to use them responsibly.

Although these legislative efforts deserve attention, they remain the exception. Only 11 states propose bills involving clinician oversight or licensure frameworks in this sample, underscoring a striking lack of regulatory engagement with the clinical dimensions of MH-AI. While many laws regulate AI in abstract or consumer-oriented terms, most fail to incorporate clinical or patient input to reflect the realities of therapeutic deployment. To address these challenges, MH-AI policy must move beyond treating clinicians as the default point of oversight and instead develop layered governance models that include professional boards, technologists, patient advocacy groups, and public regulators in shared accountability structures.

Key lessons to take away are that effective MH-AI oversight should (1) be layered and role-based with developers, deployers, clinicians, and boards each carrying defined duties calibrated to risks; (2) account for unlicensed consumer platforms and practitioners; (3) involve in some degree licensing boards and invest them with real authority; and (4) avoid offloading unworkable validation standards onto the point-of-care and instead determine the best point in the process for capability-appropriate audits.

### Harm

A small number of state legislatures have begun to confront the potential harms of MH-AI, introducing proposals focused on liability allocation, harm prevention and crisis response standards, safeguards for vulnerable users, fraud protections, and limited exemptions for research and therapeutic use.

For example, most states have yet to clarify how malpractice and liability laws specifically apply to MH-AI, leaving uncertainty about how courts might classify such technologies, whether as products, clinical tools, or services subject to standard-of-care analysis. Only a few states, most notably California and New York, explicitly proposed legislation that establishes specific claims for damages related to AI-induced harms, such as self-harm or suicide [54-57], although a few other states propose a more general AI-harm liability framework (eg, Missouri HB 1462, “Any direct or indirect harm caused by an AI system’s operation, output, or recommendation…shall be the responsibility of the owner or user who directed or employed the AI.”) [24]. New York and Rhode Island both introduce strict liability (ie, liability regardless of negligence or intent) for harm under certain circumstances [56,58]. Both California and Rhode Island also introduce affirmative defenses (ie, a defense even if one is legally responsible for the harm): California SB 813 provides an affirmative defense linked to compliance with certification standards, while Rhode Island SB 358 grants an affirmative defense to strict liability if the model “satisfied the standard of care applicable to humans who perform the same function” [48,58]. Conversely, North Carolina’s HB 934 immunizes developers entirely, assigning liability solely to clinicians—a concerning policy that places disproportionate responsibility on providers who may lack sufficient technological expertise to foresee or mitigate AI failures, including those introduced through problematic automatic software updates [49]. An emerging concern, then, is not simply a lack of liability rules but the effect of their asymmetry: inconsistent provisions risk overassigning responsibility to clinicians while underspecifying developer and deployer duties.

Only 16 of the 143 reviewed bills directly addressed malpractice and liability allocation, with just one enacted within the study period (Appendix S2 in Multimedia Appendix 1). This scarcity signals significant policy gaps, increasing risks of inconsistent liability outcomes. Oklahoma’s HB 1915 illustrates another liability-related challenge by mandating that developers track and document clinicians’ overrides of AI recommendations, thereby creating potential evidence in malpractice litigation [28]. Such provisions underscore the need for more transparent standards and clinician engagement regarding the acceptable use of AI in clinical practice, as well as how that use will influence both the standard of care and associated medicolegal risks.

Regarding safety standards, 52 bills addressed harm prevention, of which 8 bills pertained to crisis response protocols and 15 bills addressed exemptions to other provisions (eg, data privacy restrictions) to prevent imminent harm. California SB 243 and New York AB 6767, for example, explicitly mandate crisis intervention plans for detecting suicidal ideation, though incident reporting mechanisms remain uncommon [24,54,57,59-68]. California’s SB 243 notably requires annual reporting on suicidal ideation incidents but without mandating public disclosure, thus limiting transparency [54]. Missouri’s HB 1462 requires specifically “owners or developers” of AI systems involved in incidents resulting in bodily harm or death to “promptly notify the relevant authorities,” but creates no special system for doing so [24].

Risk stratification frameworks proposed in several states significantly influence MH-AI oversight, typically centering on “high-risk” AI systems used in “consequential decisions” impacting fundamental services, legal rights, and health care. Oklahoma’s HB 1916 introduces a formally tiered risk categorization, labeling health care AI as high-risk and subjecting it to stringent oversight [27]. However, definitions of scope remain unclear, raising questions about how specific applications (eg, cognitive behavioral therapy chatbots) should be categorized. At least 33 bills reviewed suggest a potential growing consensus around heightened scrutiny for MH-AI systems by placing them in the highest risk categories, although practical definitions remain ambiguous, and only 5 had been enacted at the time of the sample. In short, a few states aspire toward “high-risk” frameworks and crisis protocols, but even in those cases, rarely define operational triggers, minimum controls, or reporting expectations. Absent concrete thresholds (eg, suicide-risk detection, human-override requirements, and postmarket surveillance), “high-risk” may come to embody a simple administrative hurdle or—more concerningly—a label rather than a true safety regime.

One of the most common harms addressed in these bills was the threat of deceit, fraud, or abuse in a consumer protection context, with 84 of the bills identified addressing these topics, whether directly by creating new standards or incorporating AI systems with existing state consumer protection laws (39 bills). Bills out of Nevada, New Jersey, California, and Utah explicitly prohibit AI systems from impersonating licensed clinicians or advertising without clear disclosures (Nevada AB 406, New Jersey SB 4463, California AB 489, and Utah HB 452) [22,36,45,66], joining a total of 14 bills addressing advertising related to covered AI systems [22,33,36,66,69-78].

Protections for vulnerable populations also remain uneven. Although 50 bills addressed responsibilities toward vulnerable groups generally, the vast majority (n=39) appear focused on protections for minors (eg, California AB 1064 [79] and Nevada AB 406 [36]), with none explicitly considering risks to users with limited English proficiency, cognitive impairments, or severe mental illnesses. However, some of these categories might fall under otherwise protected disability categories. Similarly, only 1 of the 143 bills reviewed addresses mandatory reporting obligations for MH-AI operators upon detecting signs of abuse or neglect, highlighting a potentially significant oversight and underscoring the tension between patient safety and practical feasibility for AI developers [29].

This may illustrate that the immediate legislative impulse has been to regulate impersonation and deception while leaving untouched more difficult problems concerning manipulative designs, biased recommendations, undisclosed commercial influence, and misuse targeting vulnerable populations. For example, it is not difficult to imagine an AI-supported chatbot, diagnostic tool, report generator, or other MH-AI system that systematically makes recommendations or uses language in support of a particular product or service. Simultaneously, protections cluster around minors while leaving other at-risk users (eg, limited English proficiency, cognitively impaired, and severely mentally impaired) thinly served.

Finally, despite 45 bills containing provisions exempting or touching on aspects of legitimate research use, all bills sampled appear to lack clear protections or exemptions for the legitimate use of MH-AI in research and treatment involving techniques such as behavioral influence or limited disclosure. By contrast, the European Union’s AI Act (Article 5) includes targeted exemptions for approved uses involving forms of persuasion, manipulation, or partial information to achieve clinically valid outcomes [80]. Without similar carve-outs, well-intentioned bills like Montana HB 178, which broadly prohibit “cognitive behavioral manipulation of a person or group,” may unintentionally limit future innovation and care in the mental health space [81].

Key lessons to take away are that durable harm prevention in MH-AI requires (1) balanced liability that reaches developers, deployers, and clinicians; (2) specified safety baselines for high-risk uses (eg, detection, escalation, human-in-the-loop, and incident reporting) and populations; and (3) safeguards that target manipulative or biased design without foreclosing clinically legitimate influence in treatment or research.

### Autonomy

As MH-AI systems increasingly intersect with patient care, user autonomy has emerged as a critical regulatory and ethical priority, with state legislation primarily addressing transparency, disclosure, and informed consent.

Legislative efforts around transparency remain limited, with relatively few states proposing public registries or disclosure standards specifically targeting MH-AI systems. Of the 91 bills identified addressing transparency broadly, 19 explicitly mandate public-facing inventories or similar public registry reporting requirements (eg, Pennsylvania HB 290, Georgia HB 988, Delaware HB 333, Illinois HB 3529, and Illinois HB 3720) [82-86]. California’s SB 813 uniquely integrates transparency benchmarks within its voluntary certification system [48]. Despite these initiatives, transparency mandates tailored specifically for MH-AI remain uncommon, leaving clinicians and patients reliant on proprietary technologies that are not required to provide training or safety data, validation or outcome data, or other information arguably necessary for meaningful disclosure and consent.

Disclosure requirements are among the most frequently proposed regulatory strategies, with 96 bills addressing disclosure or consent in some form. Many explicitly mandate clear notification to users interacting with AI systems (eg, California AB 410, Indiana HB 1620, Massachusetts HB 1975, Utah SB 226, and Illinois HB 5649), and a few states, such as Nevada (SB 186) and New York (AB 6767), propose continuous or repeated disclosures in interactive settings [20,32,39,40,57,87,88]. California’s AB 3030 creates an exemption to some disclosure requirements if a licensed provider has reviewed the AI-generated output [46]. Explicit informed consent requirements for MH-AI are considerably rarer; only a small number of bills, namely Texas HB 1265, Pennsylvania SB 631, and Rhode Island HB 6285, directly require informed consent specifically for MH-AI services [29,30,89]. Notably, bills such as Massachusetts HB 1975 and Illinois HB 5649 propose providing users with the option of non-AI alternatives for services, although such provisions remain exceptions [32,39].

Without detailed statutory or professional guidelines, clinicians are left with unanswered practical questions, particularly regarding the level of understanding required of AI systems to meet disclosure and informed consent obligations. For example, should clinicians review and communicate error rates, data sources, or regulatory status in a manner comparable with how they communicate the risks, benefits, and limitations of medications or other clinical interventions? If the law protects such information as proprietary or fails to promote transparency or external validation of clinical MH-AI, it also raises the question of where responsibility—and liability—for harm should fall. Without clear legislative or professional guidelines, clinicians face potential liability risks, and patient autonomy remains inadequately protected.

Key lessons include that effective MH-AI autonomy protections should yield usable choices through (1) plain-language disclosure of AI use, limitations, and provenance; (2) consideration of rights to human review or non-AI pathways for consequential decisions; (3) development of scaling consent requirements based on risk; and (4) accounting for point-of-care limitations in expertise by facilitating policies that appropriately distribute transparency requirements.

### Privacy and Data

Despite the uniquely sensitive nature of mental health data, few states propose tailored privacy protections for MH-AI systems. Legislative efforts remain limited, focusing primarily on addressing gaps in existing privacy frameworks, ensuring user control over AI-generated data, and establishing exceptions for crisis-related interventions.

Traditional mental health records, such as psychotherapy notes and substance use treatment records, receive specialized privacy protections under HIPAA and 42 Code of Federal Regulations Part 2 [90,91]. However, none of the reviewed state bills explicitly extend similar protections to MH-AI–generated content, such as therapy-like transcripts, emotional disclosures, or sensitive behavioral metadata (excepting, perhaps, Illinois HB 1806, which brings such records under the state’s Mental Health and Developmental Disabilities Confidentiality Act, but analysis of this separate law is outside the scope of this review) [33]. Of the 77 bills addressing data protection, the lack of specialized protections may be particularly pertinent for non–HIPAA-covered entities, such as app developers and commercial platforms, creating a potential regulatory gap that leaves clinicians and patients vulnerable to data misuse and potential reidentification. Perhaps compounding the issue, several bills specifically exclude HIPAA-covered entities from their protections, relying on a federal statutory framework that also does not consider many MH-AI uses and products (eg, Rhode Island S0627 exempts HIPAA-covered entities providing health care recommendations that “are not considered to be high risk”) [23,68,69,78,92-108]. Therefore, within this snapshot of legislation, the present privacy posture is mainly entity-centric when the risk is data-centric. MH-AI generates unique data products that are sensitive regardless of who holds them.

Few state bills meaningfully address users’ rights to control their mental health data within MH-AI systems. Notable exceptions include California AB 1018 and Colorado SB 24‐205, which grant users access rights to data involved in high-risk AI decisions [72,95]. In contrast, North Carolina’s SB 624 uniquely mandates a 30-day self-destruction timeline for MH-AI chat data and imposes enhanced encryption and data reuse restrictions for such applications [60]. However, these data control provisions introduce practical challenges related to clinical documentation, adverse event review, and compliance with existing medical record retention laws—placing them in tension with clinicians’ ethical and legal obligations.

Alongside the safety exemptions mentioned above, several bills aim to strike a balance between data privacy and immediate patient safety concerns, such as Colorado SB 24‐205 and Virginia HB 747, which permit the temporary suspension of privacy rules during emergencies [95,109]. While potentially critical in crises involving self-harm or suicide risk, such exceptions raise serious concerns about intervention protocols, accountability for misuse, and access by law enforcement or the legal system. Without well-defined protocols and robust oversight, these exemptions risk undermining patient trust and clinical integrity and could disproportionately impact communities already experiencing heightened surveillance or systemic mistrust.

Key lessons include that effective MH-AI privacy frameworks should (1) be data-centric; (2) govern data lifecycle use throughout collection, inference, retention, and sharing; and (3) integrate bounded emergency exceptions, auditable controls, and rights that are workable in clinical settings.

The goal is not maximal secrecy or maximal access, but accountable use.

### Limitations

This review should be interpreted with several limitations in mind. First, the analysis represents a cross-sectional snapshot of policy content in legislation as it existed on May 19, 2025, regardless of whether each bill was later amended, enacted, or failed. This study’s intent was to characterize what states were proposing for MH-AI, but not to systematically evaluate political feasibility, legislative trajectories, or implementation effects.

Second, legislation was collected from a single source, LegiScan, a database that aggregates bills from all 50 states and presents them in a standardized format. This approach ensured consistent capture of bill texts at a defined point in time, regardless of legislative status. Such consistency was necessary given the highly dynamic nature of the legislative process: bills may exist in multiple versions in different legislative chambers, undergo repeated committee revisions, fail, and later be reintroduced under different identifiers, and are subject to state-specific procedural rules. By using a centralized platform, the analysis minimized the effects of procedural variation across states. It also enabled more direct comparisons, although it necessarily depended on the integrity and coverage of the LegiScan service. Relatedly, companion bills were treated as a single legislative item to reduce redundancy; however, this simplification may obscure substantive differences between House and Senate versions of some bills.

Third, the search strategy relied on keyword-based retrieval. Multiple candidate terms were evaluated, but a refined set was ultimately adopted to balance 2 objectives: narrowing the dataset to legislation (plausibly relevant to clinical AI) while excluding a large volume of unrelated bills. This compromise inevitably excluded some relevant measures, particularly those using indirect or atypical terminology. For example, North Carolina’s HB 934, noted earlier, was omitted despite its substantive relevance because it did not include qualifying keywords. More broadly, statutory frameworks are often fragmented and depend heavily on cross-references to agency regulations, definitional clauses, and related instruments. Even with comprehensive protocols, such complexity limits the ability to capture all relevant legislation. In the future, computational methods such as natural language processing might be used instead to identify relevant legislation beyond keyword-based retrieval. Nevertheless, the bills analyzed provide meaningful insight into emerging policy patterns and legislative concerns across US states.

Fourth, simple counts of bills or tags are activity metrics, not quality metrics. They do not capture scope, enforceability, or implementation (eg, funding, effective dates, sunset clauses, and exemptions). One omnibus statute can cover more policy areas than several narrow bills, while some tags reflect incidental mentions rather than operative provisions. Counts also reflect drafting style and calendar differences across states, so higher numbers may indicate legislative fragmentation rather than greater comprehensiveness or effectiveness.

### Future Directions and Recommendations

Future research should move beyond cataloging and classifying bills to more fully interrogate the substantive policy directions emerging from state legislatures. The tagging system developed for this study could be used to trace specific themes across jurisdictions over time. Such a unified approach will enable comparative analyses that highlight both convergence and divergence in state approaches, providing evidence to inform potential federal policymaking or the development of national standards.

The current findings suggest that mental health remains one of the most consequential yet least prepared frontiers of AI regulation. Legislative activity is marked by rapid growth but fragmented execution, with critical gaps in professional oversight, liability frameworks, privacy protections, and safeguards for vulnerable populations. Furthermore, clinicians are frequently assigned responsibilities without corresponding guidance or resources, effectively shifting complex regulatory burdens to the point of care. The limited inclusion of mental health practitioners or patients in legislative task forces and advisory bodies underscores the risk of policymaking that diverges from clinical realities.

Addressing these gaps will require greater engagement from mental health professionals, institutions, and professional associations. Active collaboration with policymakers and patient advocacy groups is necessary to ensure that regulatory frameworks are clinically informed, ethically grounded, and practically feasible. To facilitate this process, the authors provide (1) Appendix S3 in Multimedia Appendix 1, which proposes a scoring framework for interpreting state engagement across key legal domains relevant to MH-AI and is less reliant on the simple counting scheme used in this analysis with its previously mentioned limitations; and (2) access to the tagging database of MH-AI related bills created for this study [110]. By raising awareness of MH-AI policy issues and providing practical tools, this work lays the groundwork for more active clinician engagement in shaping the next generation of AI regulation, whether through direct advocacy or further research. Clinician and patient involvement at this juncture will be pivotal in determining whether MH-AI governance advances care, safety, and innovation.

### Conclusion

This 50-state review demonstrates that while legislative activity related to MH-AI is widespread, it remains uneven. Although hundreds of AI-related bills have been introduced since 2022, only a small fraction explicitly address mental health, and enacted provisions remain limited in scope. Where regulation exists, it is fragmented across states and typically incorporates mental health incidentally rather than as a distinct policy focus.

Across jurisdictions, 4 recurring domains emerge: professional oversight, harm prevention, patient autonomy, and data governance. These domains reflect central questions raised by MH-AI but are addressed inconsistently, with significant variability in scope and emphasis. Gaps remain evident within each domain, such as how liability is allocated between developers and clinicians, how adverse events are reported, and how sensitive data are protected. In addition, while this review highlights proposed provisions of law, omissions may carry equal or greater significance.

Taken together, these findings demonstrate that state legislatures are actively shaping the regulatory environment for MH-AI, but in fragmented and incomplete ways. This evolving regulatory landscape is characterized by sporadic attention to mental health, limited integration of clinical perspectives, and reliance on broadly framed statutes that may fail to capture the realities of psychiatric practice. This analysis establishes a baseline for tracking future policy development and assessing whether subsequent legislation achieves greater coherence, specificity, and responsiveness to the complexities of mental health care.

## Supplementary material

10.2196/80739 Multimedia Appendix 1 Tables of taxonomy coding outcomes, enacted bill data with figures, and state Mental Health AI Regulatory Coverage Index Scoring Framework.

## Abbreviations

AB: Assembly Bill
AI: artificial intelligence
HB: House Bill
HIPAA: Health Insurance Portability and Accountability Act
II: Incidentally Implicative
MH-AI: mental health–related artificial intelligence
NR: Not Relevant
SB: Senate Bill
SR: Substantively Relevant
