# Supplementary material for: Governing AI in Mental Health: 50-State Legislative Review
Source: JMIR Ment Health. 2025 Oct 31;12:e80739. doi: 10.2196/80739 (PMC12578431; doi:10.2196/80739)
Supplement: Multimedia Appendix 1 [file mental-v12-e80739-s001.docx]

# Appendix S1: Taxonomy Coding Outcomes

**Appendix Exhibit S1 (Table S1).** Number of bills of each taxonomy type by state.

|  | E | SR | II | Total |  |  | E | SR | II | Total |
| --- | --- | --- | --- | --- | --- | --- | --- | --- | --- | --- |
| Total | 28 | 79 | 36 | 143 |  | PA | 1 | 0 | 0 | 1 |
| CA | 6 | 5 | 8 | 19 |  | NC | 1 | 0 | 0 | 1 |
| NY | 3 | 7 | 4 | 14 |  | IN | 1 | 0 | 0 | 1 |
| IL | 8 | 1 | 0 | 9 |  | MT | 0 | 1 | 0 | 1 |
| TX | 1 | 6 | 2 | 9 |  | ME | 0 | 1 | 0 | 1 |
| MA | 1 | 5 | 2 | 8 |  | MS | 0 | 1 | 0 | 1 |
| RI | 1 | 5 | 2 | 8 |  | OH | 0 | 0 | 1 | 1 |
| OK | 0 | 5 | 2 | 7 |  | AK | 0 | 0 | 1 | 1 |
| MD | 0 | 5 | 2 | 7 |  | ND | 0 | 0 | 1 | 1 |
| HI | 0 | 5 | 1 | 6 |  | FL | 0 | 0 | 1 | 1 |
| VA | 0 | 5 | 1 | 6 |  | KY | 0 | 0 | 1 | 1 |
| NM | 1 | 2 | 1 | 4 |  | MO | 0 | 0 | 1 | 1 |
| WA | 0 | 3 | 1 | 4 |  | AL | 0 | 0 | 1 | 1 |
| NV | 1 | 2 | 0 | 3 |  | OR | 0 | 0 | 0 | 0 |
| NJ | 1 | 2 | 0 | 3 |  | MI | 0 | 0 | 0 | 0 |
| AR | 0 | 3 | 0 | 3 |  | KS | 0 | 0 | 0 | 0 |
| UT | 2 | 0 | 0 | 2 |  | TN | 0 | 0 | 0 | 0 |
| MN | 0 | 2 | 0 | 2 |  | ID | 0 | 0 | 0 | 0 |
| SC | 0 | 2 | 0 | 2 |  | IA | 0 | 0 | 0 | 0 |
| LA | 0 | 2 | 0 | 2 |  | DE | 0 | 0 | 0 | 0 |
| GA | 0 | 2 | 0 | 2 |  | AZ | 0 | 0 | 0 | 0 |
| VT | 0 | 2 | 0 | 2 |  | WI | 0 | 0 | 0 | 0 |
| CT | 0 | 2 | 0 | 2 |  | WV | 0 | 0 | 0 | 0 |
| CO | 0 | 2 | 0 | 2 |  | WY | 0 | 0 | 0 | 0 |
| NE | 0 | 1 | 1 | 2 |  | SD | 0 | 0 | 0 | 0 |
| NH | 0 | 0 | 2 | 2 |  |  |  |  |  |  |

**Appendix Exhibit S2 (Table S2).** Number of enacted bills of each taxonomy type by state.

|  | **E** | **SR** | **II** | **Total** |
| --- | --- | --- | --- | --- |
| Total | 3 | 10 | 7 | 20 |
| CA | 0 | 2 | 3 | 5 |
| UT | 2 | 0 | 0 | 2 |
| NY | 1 | 0 | 1 | 2 |
| NH | 0 | 0 | 2 | 2 |
| MD | 0 | 1 | 0 | 1 |
| WA | 0 | 1 | 0 | 1 |
| VA | 0 | 1 | 0 | 1 |
| ND | 0 | 0 | 1 | 1 |
| NM | 0 | 1 | 0 | 1 |
| NV | 0 | 1 | 0 | 1 |
| MT | 0 | 1 | 0 | 1 |

# Appendix S2: Enacted Bill Data

**Appendix Exhibit S3 (table S3).** Tags among enacted bills only.

| Tag | Number of Bills |
| --- | --- |
| Consumer Protection | 12 |
| Transparency | 12 |
| Disclosure/Consent | 12 |
| Civil Penalties | 10 |
| Data Protection | 9 |
| Discrimination/Bias | 8 |
| Meta/Biometric Data | 7 |
| Research | 7 |
| Post-Market Review | 6 |
| Monitoring | 6 |
| Vulnerable Populations | 5 |
| Risk Classification | 5 |
| Practitioner Responsibilities | 4 |
| Safety Standards | 4 |
| Licensing Board Oversight | 3 |
| Special Purpose Entities | 3 |
| Human-in-the-Loop | 3 |
| Criminal Penalties | 2 |
| Opt Out | 2 |
| Event Reporting | 2 |
| Pilot/Sandbox | 1 |
| Malpractice | 1 |
| Pre-Market Review | 0 |
| Payments/Insurance | 0 |
| Prescribing | 0 |


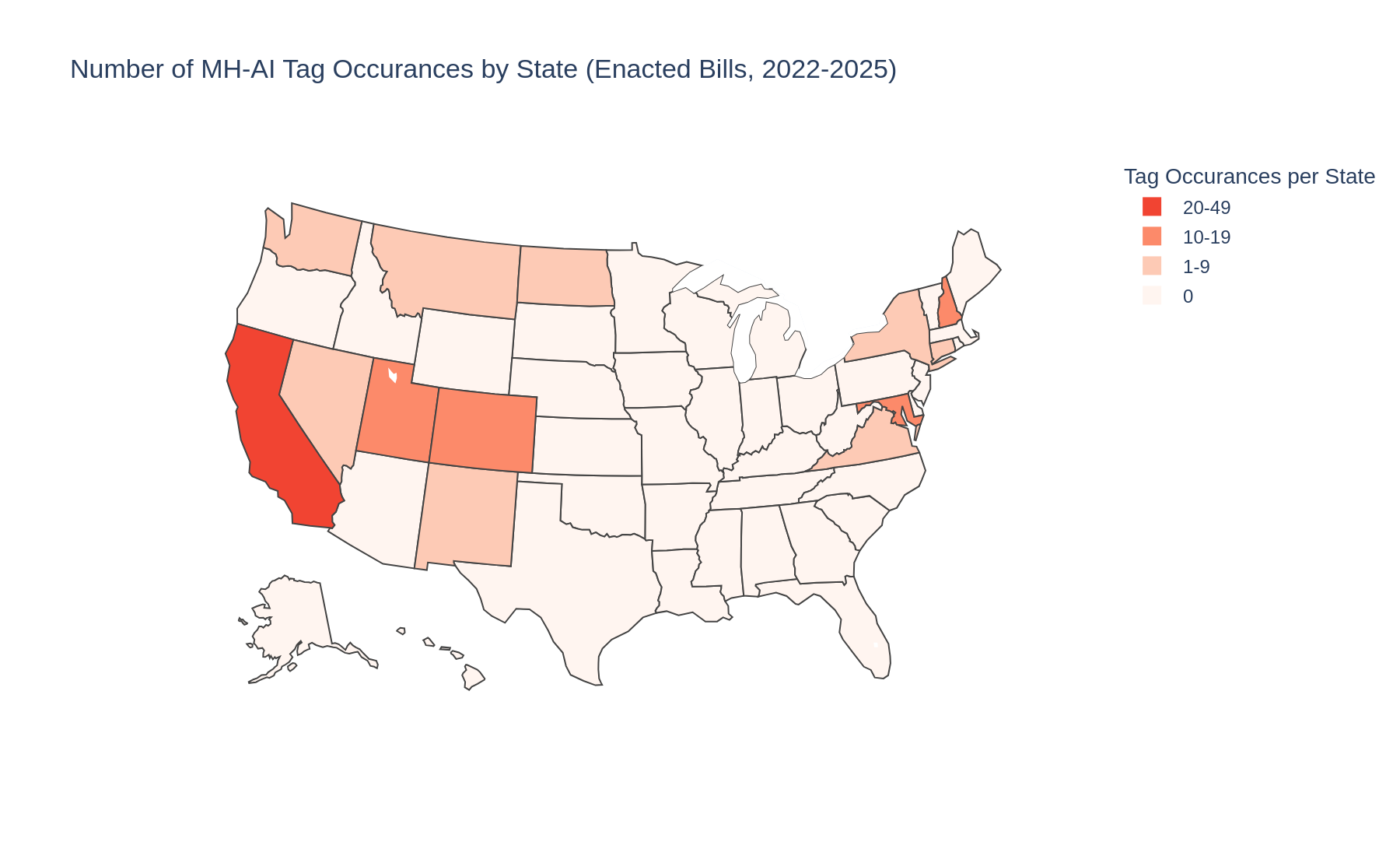


**Appendix Exhibit S4 (Figure S1, above).** Number of total tag occurrences in enacted bills by state (2022-2025).


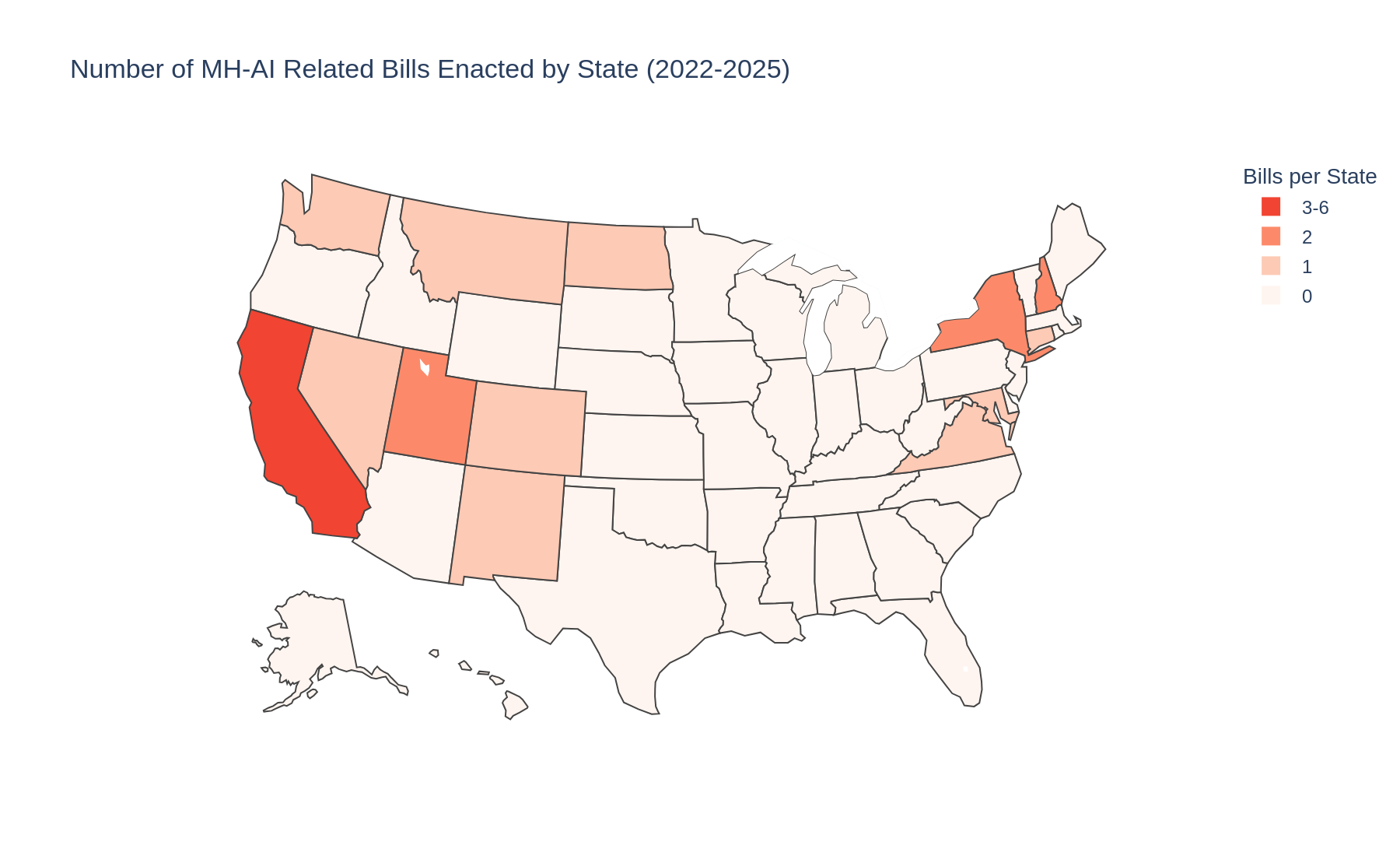


**Appendix Exhibit S5 (Figure S2, above).** Number of Enacted MH-AI bills by U.S. State (2022-2025).


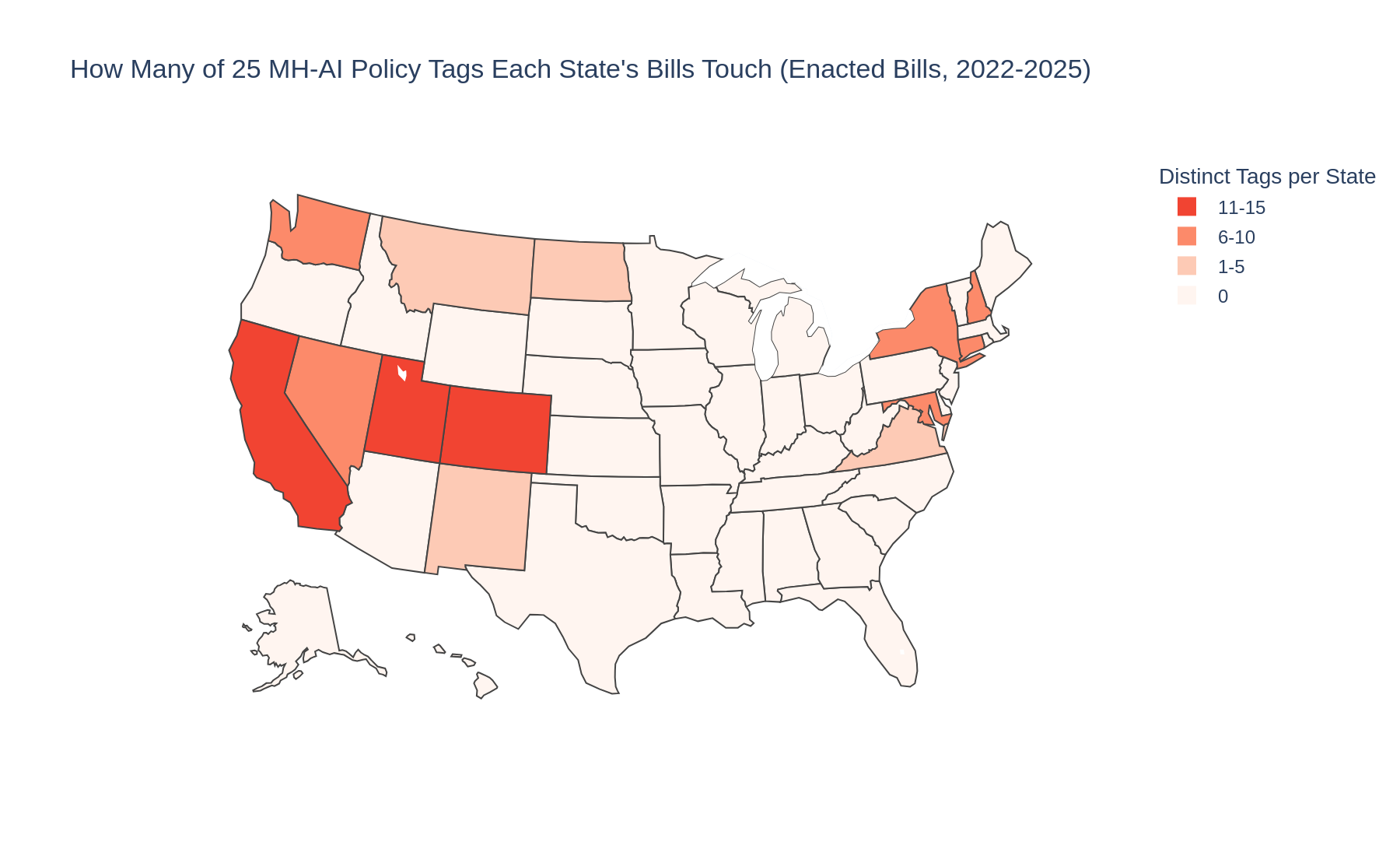


**Appendix Exhibit S6 (Figure S3, above).** Number of tags out of 25 possible identified in enacted MH-AI bills by U.S. State (2022-2025).

# Appendix S3: State MH-AI Regulatory Coverage Index

State approaches to regulating MH-AI vary widely in scope and focus. To illustrate this variation, the authors developed a State MH-AI Regulatory Coverage Index (RCI), which could be used to score each state based on the extent to which its **enacted** laws address key legal domains relevant to the clinical use of MH-AI.

Importantly, RCI scores are intended to describe the breadth of legal engagement and are not meant to suggest that higher or lower scores are inherently better or worse. For example, a state with a more *permissive* outlook that does not require clinician oversight of MH-AI tools may score “0” in that domain, while a more *restrictive* framework might receive a higher score. State laws may also leave seeming gaps where they expect other regulatory schemes, such as federal law or regulations, to fill those areas. The index is descriptive rather than evaluative and should be interpreted accordingly.

Finally, while the authors considered attempting to apply the RCI to an example case, ultimately this is an exercise best left to policy experts in each respective state who are familiar with the state’s case law, state regulatory agency guidelines, state health laws, and other aspects of the state’s particular regulatory scheme not captured by the limited search protocol used in this study.

**Appendix Exhibit S7 (Table S4, above).** State Mental Health AI Regulatory Coverage Index scoresheet.

| **Mental Health AI Regulatory Coverage Index (MH-AI RCI) Scoring Framework** | | |
| --- | --- | --- |
| **Category** | **Points Available** | **Scoring** |
| **Explicit MH-AI Coverage** | 0-2 | MH-AI is clearly addressed in one or more bills. Additional point for comprehensive framework. |
| **Clinician Oversight** | 0-2 | Addresses licensed supervision and/or licensing board roles. Additional point for requiring a mental health professional or representative on pertinent task forces/committees. |
| **Safety & Incident Protocols** | 0-1 | Addresses suicide risk detection, crisis response, real-time monitoring, or incident reporting. |
| **Autonomy Protections** | 0-1 | Addresses transparency, informed consent, opt-out alternatives, or AI disclosures. |
| **Data Privacy & User Control** | 0-1 | Addresses protections (and exemptions) for MH-AI related data and user data control. |
| **Risk Tiering** | 0-1 | Addresses the comparative risk between different types of MH-AI systems. |
| **Legitimate Uses** | 0-1 | Addresses exemptions or protections for legitimate treatment or research uses of MH-AI. |
| **Consumer-Facing Tools** | 0-1 | Addresses consumer-facing MH-AI tools. |
| **Documentation** | 0-1 | Addresses documentation requirements or records retention related to MH-AI. |
| **Accessibility** | 0-1 | Addresses access by those with disabilities or language limitations. |
| **Transparency** | 0-2 | Addresses public access to audits, reports, and other data needed for informed use. Additional point for addressing data needed for external validation of MH-AI technologies. |
| **Misuse** | 0-2 | Addresses deceitful, fraudulent, or manipulative uses of MH-AI. Additional point for addressing advertising. |
| **Malpractice & Liability** | 0-2 | Addresses MH-AI’s role in malpractice and liability frameworks. Additional point for addressing the use of MH-AI information in litigation. |

**Maximum Score: 18**
